# Interaction between iron/folic acid and malaria

**DOI:** 10.1186/1475-2875-11-S1-O25

**Published:** 2012-10-15

**Authors:** Klaus Kraemer, Hans Verhoef

**Affiliations:** 1 Sight and Life, Basel, Switzerland; 2 London School of Hygiene and Tropical Medicine, UK; 3 Wageningen University, The Netherlands

## 

This abstract is submitted as part of the round-table discussion sponsored by Sight and Life.

A recent trial reinforced earlier concerns that iron supplementation can increase malaria rates. The World Health Organization subsequently restricted its recommendations in malaria-endemic areas from universal supplementation to targeted supplementation of iron-deficient children, but continues to advocate universal supplementation in pregnancy. Resurgent interest in iron has led to further studies to assess its safety, particularly in pregnant women; to identify markers for rapid, low-cost screening for deficiency; and to develop safe but efficacious iron interventions.

The hepcidin-axis recently emerged as a newly discovered arm of the innate immune system. Hepcidin is now known to regulate iron absorption and metabolism, but also to mediate impaired recycling and absorption of iron in infections. Current evidence suggests that plasma hepcidin concentration may predict haematological and infectious responses to iron, at least in the short term, and that deliberately altering hepcidin concentrations may result in new strategies to control infections by *Plasmodium* and other iron-requiring pathogens.

Folic acid supplementation continues to be recommended around conception and during pregnancy, and international agencies have recommended additional measures to increase the intake of folic acid. *Plasmodium* parasites can utilise exogenous folate, however, and several trials suggest that folic acid supplementation can reduce the efficacy of antifolate drugs used for malaria control. A better understanding is required of the controversies of interventions to increase folate status and the safety of interventions to improve folate status in malaria-endemic countries.

